# Financial Rewards for Smoking Cessation During Pregnancy and Birth Weight: A Meta-Analysis

**DOI:** 10.1001/jamanetworkopen.2025.0214

**Published:** 2025-03-06

**Authors:** David Tappin, Jiyoung Lee, Alex McConnachie, Loren Kock, Stephen T. Higgins, Sarah H. Heil, Ivan Berlin, Steven J. Ondersma, Frank Kee, Ira Bernstein, Linda Bauld

**Affiliations:** 1 Child Health, School of Medicine, University of Glasgow, Glasgow, United Kingdom; 2 Robertson Centre for Biostatistics, School of Health and Wellbeing, University of Glasgow, Glasgow, United Kingdom; 3 University College London, University of London, London, United Kingdom; 4 Center on Behavior and Health, Department of Psychiatry, University of Vermont, Burlington; 5 Vermont Center on Behavior and Health, University of Vermont, Burlington; 6 Département de Pharmacologie Médical, Hôpital Pitié-Salpêtrière-Sorbonne Université, Paris, France; 7 Charles Stewart Mott Department of Public Health, Michigan State University, Flint; 8 Centre for Public Health, Queen’s University Belfast, Northern Ireland, United Kingdom; 9 Department of Obstetrics, Gynecology and Reproductive Sciences, University of Vermont Medical Center, Burlington; 10 Usher Institute and SPECTRUM Consortium, University of Edinburgh, Edinburgh, United Kingdom

## Abstract

**Importance:**

Offering pregnant women financial rewards to stop smoking is associated with a more than 2-fold increase in smoking cessation and is cost-effective; however, it is possible that the association is the result of gaming of the outcome measure (eg, not smoking for 24 hours before outcome measurement using a carbon monoxide breath test). Birth weight is an outcome measure that is independent of the rewards process.

**Objective:**

To examine birth weight change associated with offering financial rewards for smoking cessation to pregnant women, and to estimate the average expected birth weight change for neonates born to those who quit smoking because of the rewards.

**Data Sources:**

Medline, American Psychological Association PsycInfo, Embase, Cochrane (the Cochrane Central Register of Controlled Trials, the Cochrane Tobacco Addiction Group Specialized Register, and the Cochrane Database of Systematic Reviews), and PubMed were searched from their inception until December 5, 2023, for published reports of trials of incentives for abstinence from substance use among pregnant women.

**Study Selection:**

Only trials using an experimental design allowing treatment effects to be attributed to the reward intervention were included. For this review update, potentially relevant studies retrieved were screened by a reviewer.

**Data Extraction and Synthesis:**

Data were pooled using both fixed-effects and random-effects models. The Preferred Reporting Items for Systematic Reviews and Meta-Analyses (PRISMA) guideline was followed.

**Main Outcomes and Measures:**

The primary outcomes were mean (SD) birth weight, birth weight for gestational age *z* score, and numbers of newborns with low birth weight (<2.5 kg) and who were small for gestational age (<10th percentile).

**Results:**

Fixed-effects intention-to-treat analysis including 8 trials (2351 participants)—2 trials from the UK (1475 participants), 1 trial from France (407 participants), and 5 trials from the US (469 participants)—estimated a statistically significant mean 46.30 g (95% CI, 0.05 to 92.60 g) birth weight increase associated with adding financial rewards for smoking cessation. Complier average causal effects analysis (2239 participants) estimated a mean 206.00 g (95% CI, −69.12 to 481.14 g) increase for neonates born to smokers who quit because of rewards, but the increase was not statistically significant. There was no association with low birth weight or birth weight adjusted for gestational age, although fewer neonates were born small for gestational age, particularly if cessation was associated with rewards (complier average causal effects risk difference −17.70%; 95% CI, −34.90% to −0.42%).

**Conclusions and Relevance:**

In this meta-analysis, neonates were significantly heavier when pregnant women were offered the addition of financial rewards contingent on smoking cessation, supporting the effectiveness and, therefore, cost-effectiveness of financial rewards in this health care setting.

## Key Points

**Question:**

Are financial rewards to encourage smoking cessation during pregnancy associated with increased neonate birth weight?

**Findings:**

In this meta-analysis of 8 randomized clinical trials from the UK, France, and the US, with 2351 participants, the addition of financial rewards contingent on smoking cessation in addition to usual stop smoking support was associated with a statistically significant population increase in birth weight of 46.30 g.

**Meaning:**

This birth weight improvement implies a real cost-effective increase in smoking cessation when women are offered the addition of financial rewards to stop smoking during pregnancy.

## Teaser

This meta-analysis examines birth weight change associated with financial rewards for smoking cessation for pregnant women.

## Introduction

Smoking throughout pregnancy is one of the most damaging behaviors affecting the fetus and is associated with a 10% decrease in birth weight (mean, 387 g)^1^ for consistent smoking and many other short-term and long-term problems,^2,3^ as well as large additional health service costs.^4,5^ A 10% decrease in birth weight in particular is associated with being small for gestational age (SGA), a physical marker of substantial and often long-term damage.^6^ Avoidance of this birth weight reduction caused by smoking would be a worthwhile and clinically effective intervention that could help convince policymakers to implement financial rewards to help pregnant women to stop smoking.

The addition of financial rewards is the most effective intervention to improve effectiveness of stop smoking services (SSS) for pregnant women.^7,8^ In this article, *SSS* is used as a generic term for any support given to help women stop smoking during pregnancy. Generally, only those compliant with stopping smoking during pregnancy receive financial rewards. More than half of the trials^8^ also reported birth weight, with most showing a nonsignificant increase in birth weight for those offered financial rewards for smoking cessation compared with no reward. This study aims to assess whether the addition of financial rewards is associated with an important increase in neonate birth weight compared with usual care alone.

Consistent smoking during pregnancy is associated with an average 387-g reduction in birth weight,^1^ which is much greater than the mean improvement in birth weight found in trials of financial rewards. An example is the Cessation in Pregnancy Incentives Trial (CPIT) II,^9^ where the improvement was 21 g. This difference was largely related to compliance. Adding conditional rewards for smoking cessation in pregnancy are examples of encouragement trials. All participants are free to stop smoking or not, but those randomized to intervention receive additional encouragement through the offer of a financial reward if successful. For some people, the intervention makes no difference, and they cannot stop either with or without this encouragement. At the other end of the spectrum are those who can quit without additional encouragement, and although the intervention results in a reward, it actually has no association with their ability to stop. Only those in the middle, who are not able to quit without the intervention, but are able to do so with additional encouragement, are affected by the intervention. Logically, it is only this group who stand to achieve downstream health benefits, for example in terms of their child’s birth weight.

Complier average causal effects (CACE) analysis can be used to estimate the association of stopping smoking with birth weight.^10,11,12^ In a randomized encouragement trial, CACE analysis estimates the association of the behavior change (stopping smoking) with the outcome (birth weight) in those people who achieve the behavior change (smoking cessation) only as a result of the randomized encouragement intervention (the offer of financial rewards).

In the trial by Tappin et al^9^ in 2015, the proportion of pregnant participants who quit smoking toward the end of pregnancy was 8.6% in the usual SSS control group and increased to 22.5% with the additional offer of financial rewards for smoking cessation. CACE analysis^10,11,12^ indicated that the small birth weight improvement of 21 g in those offered financial rewards as well usual SSS support compared with those offered usual care alone, translated into a 154 g (95% CI, −617 to 803 g; approximately 5% of birth weight) improvement for those women who quit smoking but would not have managed without the additional offer of financial rewards. However, the overall 21-g increase in birth weight and this clinically important 154-g increase among those affected did not reach statistical significance and have therefore largely been ignored by clinicians and policymakers.

This current article extends the systematic review by Kock et al^8^ and focuses the outcome on birth weight when the offer of financial rewards for smoking cessation is added to routine SSS support for pregnant women. All corresponding authors for studies in the Kock et al^8^ review and update were invited to provide additional data to allow a meta-analysis of the population-level association between the offer of financial rewards with change in birth weight, and of the associated effect of smoking cessation in the subset of women who were able to quit as a result of the intervention.

This study addresses 2 research questions. First, do neonates born to women who smoke in early pregnancy have an associated increase in mean birth weight and mean birth weight for gestational age *z* score when women are offered financial rewards contingent on quitting smoking, as well as usual SSS support compared with neonates born to mothers offered usual SSS support alone or rewards not contingent on smoking cessation (intention-to-treat [ITT] meta-analysis)? Second, what is the mean birth weight difference associated with quitting smoking during pregnancy because of the offer of financial rewards? How is this reflected in numbers of neonates born low birth weight (<2500 g) and SGA (<10th percentile)?

## Methods

We performed a systematic review and meta-analyses of randomized clinical trials examining the association between contingent financial rewards for smoking cessation during pregnancy and birth weight. This study is based on the systematic review by Kock et al.^8^ The study is reported according to Preferred Reporting Items for Systematic Reviews and Meta-Analyses (PRISMA) reporting guidelines.^13^ The study protocol was registered on PROSPERO (CRD42024494262) and has been published previously.^14^ The protocol was reviewed by West of Scotland National Health Service research ethics manager January 3, 2024, and full application was submitted to the College of Medical, Veterinary and Life Sciences ethics committee at Glasgow University on March 20, 2024. Both determined that the study could go ahead. Patient and public involvement were not undertaken for this updated systematic review and meta-analysis. Informed consent was not needed because individual patient data were not analyzed and data received from individual trial groups were fully anonymized, per the policy of Glasgow University, the center undertaking the meta-analysis.

### Protocol

Changes were made to the protocol before data were collected after discussion with trial lead authors from Kock et al.^8^ Low birth weight (<2.5 kg) and SGA were suggested as additional outcomes, and changes were made to data collection and analysis methods.

### Search Strategy and Study Selection Criteria

The search strategy and selection criteria followed those of Kock et al^8^ and were updated to allow inclusion of studies published between November 17, 2022, and December 5, 2023. Medline, American Psychological Association PsycInfo, Embase, Cochrane (the Cochrane Central Register of Controlled Trials, the Cochrane Tobacco Addiction Group Specialized Register, and the Cochrane Database of Systematic Reviews), and PubMed were searched from their inception until December 5, 2023, for published reports of trials of incentives for abstinence from substance use among pregnant women (additional details are given in the eMethods in Supplement 1). For the current review update, potentially relevant studies retrieved from the updated search were screened by Dr Kock, with detailed reasons for exclusion reported in eAppendix 1 in Supplement 1.

### Data Extraction

We reviewed studies identified by Kock et al.^8^ For studies that did not report birth weight, we contacted the corresponding authors to provide this information. Data were requested for birth weight and birth weight for gestational age *z* score (calculated by the corresponding authors using a previously published tool),^15^ including sample size (mean [SD]), the number of low-birth-weight newborns (ie, <2.5 kg), and the number of SGA newborns (ie, <10th percentile, equivalent to birth weight for gestational age *z* score less than −1.2816). For CACE analyses, these data were also collected, where available, for 4 subgroups defined by randomized group and smoking status (whether participants stopped or continued smoking).

### Statistical Analysis

The ITT associations were estimated by the differences in means (for birth weight and birth weight *z* score) and risks (for low birth weight [<2500 g] and SGA [<10th percentile]) between randomized groups. CACEs were estimated using instrumental variable regression models. The monotonicity assumption (no defiers) for CACE analysis was evaluated by examining the estimated compliance rates. ITT and CACE estimates were then pooled under both fixed-effects and random-effects models. The random-effects model used the Sidik-Jonkman^16^ method to estimate heterogeneity. The pooled association estimates were expressed as mean differences for continuous outcomes and risk differences for dichotomous outcomes, with 95% CIs. Statistical significance was defined as 95% CIs that did not include the null value of zero. Heterogeneity was examined by estimates of between study variance (τ^2^) and the *I*^2^ statistics. The possibility of publication bias was not assessed because fewer than 10 trials were included in all analyses. Cumulative meta-analyses based on publication date were conducted to evaluate evidence accumulation. Influential studies were assessed using the leave-one-out method to assess sensitivity of the overall results. Meta-analyses were done using the meta package in R statistical software version 4.2.1 (R Project for Statistical Computing).^17^ The Grading of Recommendations Assessment, Development and Evaluation (GRADE) system^18^ was used to describe strength or weakness of recommendations emanating from findings of this systematic review.

## Results

Further searches by Dr Kock to December 5, 2023, found 10 additional studies. Details of these studies and reasons all were excluded are described in eAppendix 1 in Supplement 1. The updated PRISMA diagram is in the eResults in Supplement 1. Therefore, our meta-analyses are based on trials included in the review by Kock et al^8^; 12 studies had a combined relative risk of smoking cessation toward the end of pregnancy of 2.43 (95% CI, 2.04 to 2.91). Details of the studies are available in Kock et al.^8^

Assessments of risk of bias for individual trials are in the appendix to the review by Kock et al.^8^ CACE analyses^9,19,20,21,22,23^ were subject to the availability of subgroup data, which were not available for one trial,^24^ and meeting the requirements for CACE analysis. One trial^25^ showed a negative estimated compliance rate, indicating the possible presence of defiers; therefore, this trial was excluded from CACE analyses. After examining study heterogeneity statistics, we found evidence of very low heterogeneity across all meta-analyses. As a result, the fixed-effects and random-effects models gave very similar results, and fixed-effect models are therefore reported in the figures.

The Table gives the pooled effect estimates from all fixed-effects models. eTable 1 in Supplement 1 shows corresponding results from random-effects models.

**Table.** Pooled Estimates of the Association of Smoking Cessation With Study Outcomes

Outcome and analysis	Estimate (95% CI)^a^	*P* value
Birth weight, mean difference, g		
ITT	46.30 (0.05 to 92.60)	.05
CACE	206.00 (−69.12 to 481.14)	.14
Birth weight <2.5 kg, risk difference, %		
ITT	−0.60 (−3.30 to 2.10)	.66
CACE	−3.10 (−18.55 to 12.42)	.70
Birth weight *z* score, mean difference		
ITT	0.00 (−0.12 to 0.15)	.82
CACE	0.30 (−0.38 to 0.93)	.41
Small for gestational age, risk difference, %		
ITT	−2.80 (−5.83 to 0.19)	.07
CACE	−17.70 (−34.90 to −0.42)	.04

Abbreviations: CACE, complier average causal effects estimate; ITT, intention-to-treat estimate.

^a^ Pooled estimates were derived from fixed-effects models.

### Birth Weight

**Figure 1.**
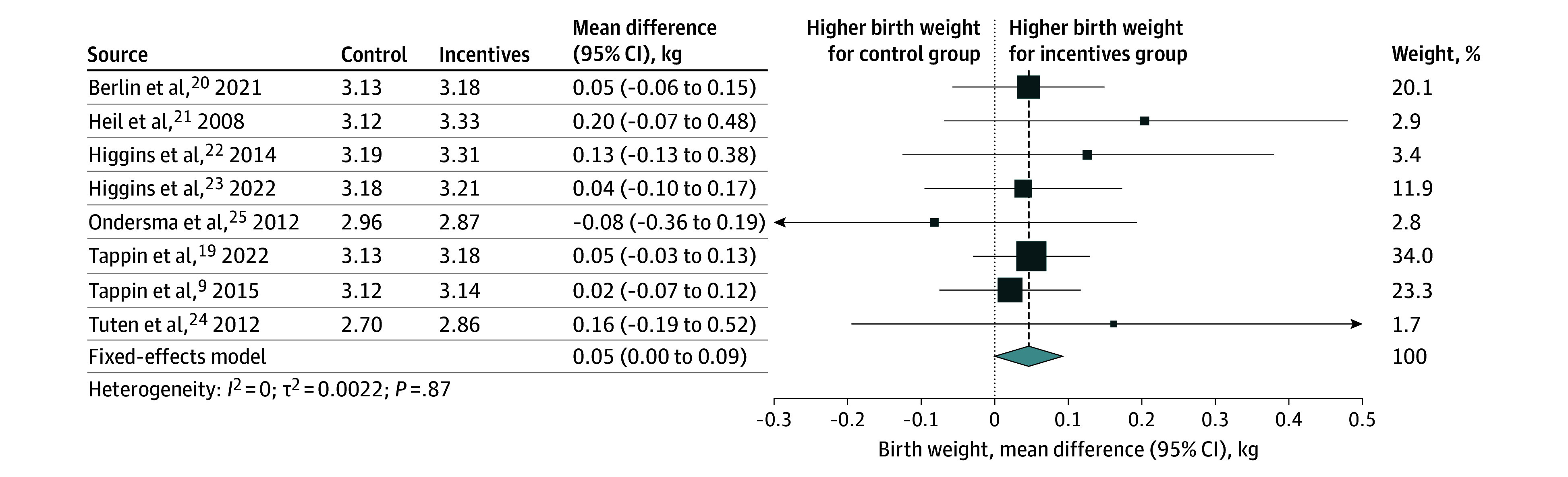
Intention-to-Treat Estimates of the Association of Financial Rewards for Smoking Cessation With Birth Weight Forest plot shows the intention-to-treat estimates of the association of the offer of financial rewards for smoking cessation during pregnancy with birth weight (kilograms). The pooled effect was calculated using a fixed-effect model. The size of data markers is proportional to the weight in the meta-analysis.

**Figure 2.**
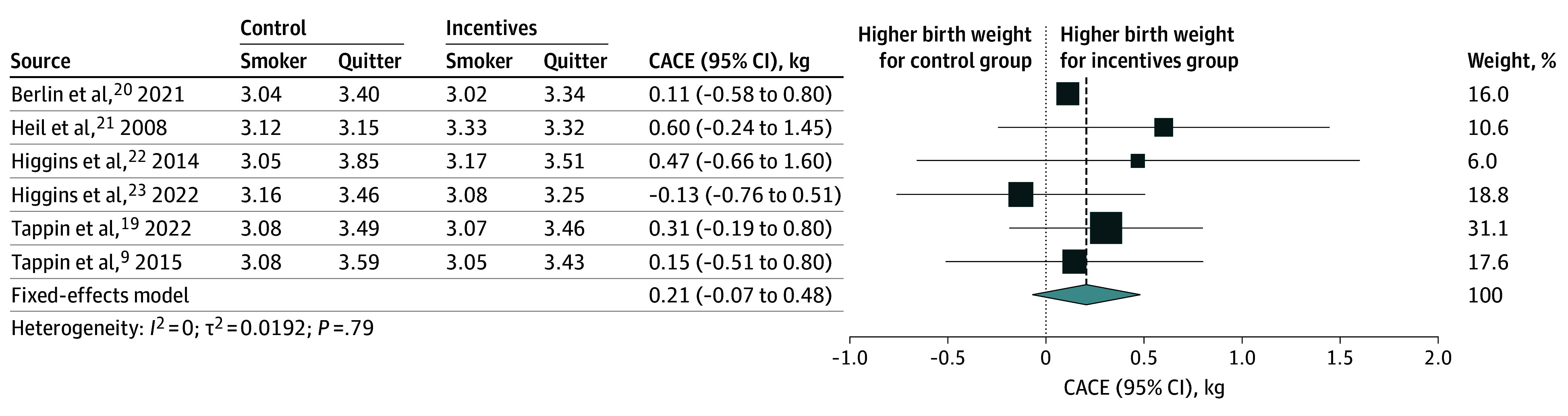
Complier Average Causal Effect (CACE) Estimates of Smoking Cessation During Pregnancy on Birth Weight Forest plot shows the CACE estimates of the association of smoking cessation during pregnancy with birth weight (kilograms). The pooled effect was calculated using a fixed-effect model. The size of data markers is proportional to the weight in the meta-analysis.

Birth weight data were available for 8 trials,^9,19,20,21,22,23,24,25^ with a total of 2351 participants: 2 trials from the UK (1475 participants),^9,19^ 1 trial from France (407 participants),^20^ and 5 trials from the US (469 participants).^21,22,23,24,25^ Pooled analysis showed that the mean birth weight of neonates born to women in the financial rewards group was 46.30 g higher (95% CI, 0.05 to 92.60 g; GRADE, moderate) compared with the control group (Figure 1 and Table). In the CACE analysis, we included 6 trials^9,19,20,21,22,23^ with subgroup data on birth weight that met required conditions for analysis. Pooled CACE estimate showed that among women who stopped smoking due to financial rewards, the mean newborn weight gain was 206.00 g (95% CI, −69.12 to 481.14 g; 2239 newborns), but the increase was not statistically significant (Figure 2 and Table). A sensitivity analysis including a trial^25^ that did not meet the monotonicity condition for CACE analysis showed similar results (eFigure 1 in Supplement 1).

The GRADE approach^18^ was used to systematically assess the certainty of evidence that birth weight increase is associated with the offer of financial rewards for smoking cessation to pregnant women (eAppendix 2 in Supplement 1). Although statistically significant, evidence for the increase in birth weight was graded as moderate owing to potential imprecision in the effect estimate (95% CI, 0.05 to 92.60 g improvement), likely related to sample size.

### Low Birth Weight (<2.5 kg)

Pooling showed there was no ITT (risk difference, −0.6%; 95 CI, −3.30% to 2.10%; 7 studies; 2300 newborns) or CACE (risk difference −3.1%; 95% CI, −18.55% to 12.42%; 6 studies; 2239 newborns) association with the risk of low birth weight. See details in eFigures 2 and 3 in Supplement 1 and the Table.

### Birth Weight for Gestational Age *z* Score and SGA

To account for variations in birth weight with gestational age and sex, we performed meta-analyses on birth weight *z* scores available from 5 trials.^20,21,22,23,25^ Pooled effect estimates showed no clear evidence of ITT or CACE association with birth weight adjusted for gestational age and sex (Table and eFigures 4 and 5 in Supplement 1).

**Figure 3.**
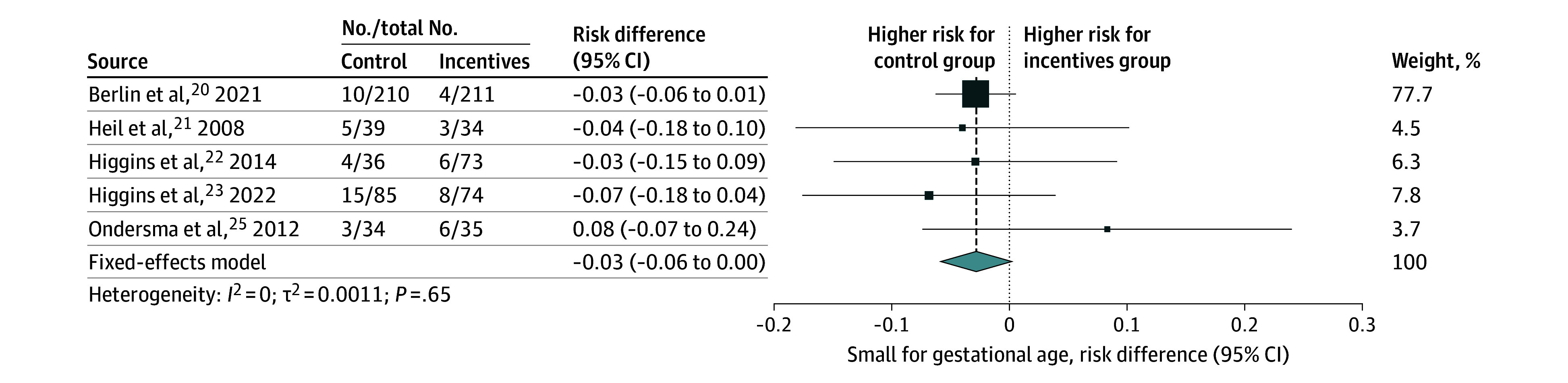
Intention-to-Treat Estimates of the Association of Financial Rewards for Smoking Cessation With Small for Gestational Age Forest plot shows the intention-to-treat estimates of the association of the offer of financial rewards for smoking cessation during pregnancy with the risk of being born small for gestational age (<10th percentile), expressed as risk differences. The pooled effect was calculated using a fixed-effect model. The size of data markers is proportional to the weight in the meta-analysis.

**Figure 4.**
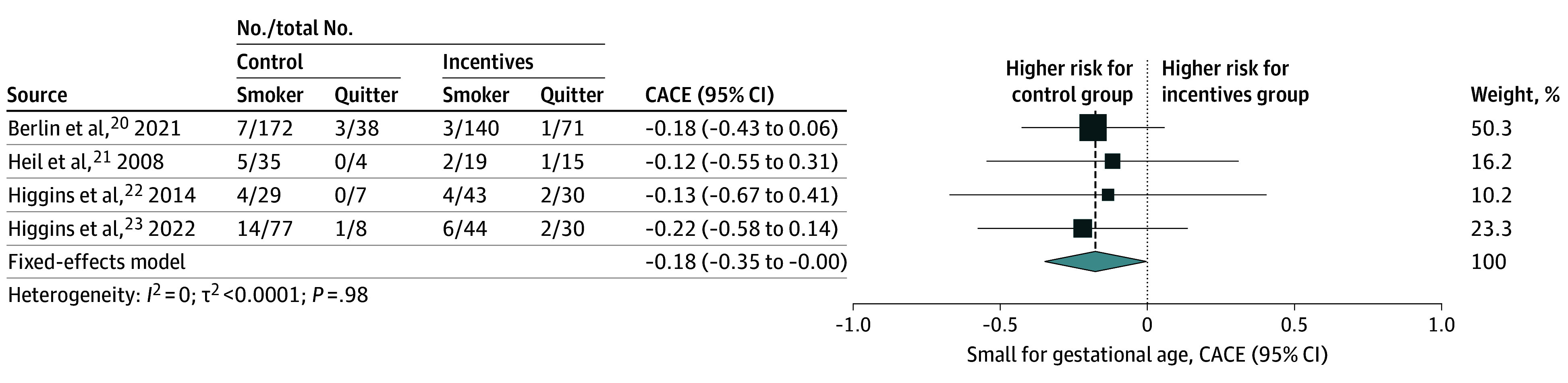
Complier Average Causal Effect (CACE) Estimates of Smoking Cessation During Pregnancy on Small for Gestational Age Forest plot shows the CACE estimates of the association of smoking cessation during pregnancy with the risk of being born small for gestational age (<10th percentile), expressed as risk differences. The pooled effect was calculated using a fixed-effect model. The size of data markers is proportional to the weight in the meta-analysis.

We also investigated the association between financial rewards and the risk of neonates born SGA (*z* score <10th percentile). Pooling showed a small but nonsignificant reduction in the associated risk of being born SGA with the offer of financial rewards (risk difference, −2.80%; 95% CI, −5.83% to 0.19%; 5 studies; 825 newborns) (Figure 3 and Table). However, according to our pooled CACE analysis, we found evidence of a significant reduction in the risk of SGA due to smoking cessation induced by the rewards among those who quit smoking as a result of the intervention (−17.70%; 95% CI, −34.90% to −0.42%; 4 studies; 755 newborns) (Figure 4 and Table).

### Sensitivity Analyses

eFigures 6 and 7 in Supplement 1 show cumulative forest plots for each analysis. As more data accrued, pooled estimates appear to have stabilized and become more precise. eFigures 8 and 9 in Supplement 1 show pooled estimates for each analysis after leaving each trial out in turn. With little heterogeneity observed overall, no individual trial appears to have an undue influence on any analysis.

##  Discussion

This systematic review and meta-analysis found a significant increase in birth weight of 46.30 g (95% CI, 0.05 to 92.60 g) associated with the offer of financial rewards to pregnant women who smoke contingent on smoking cessation compared with those offered routine SSS support alone (whatever that support may be). This result supports the primary trial outcomes that increased prolonged smoking cessation does take place during pregnancy with the additional offer of financial rewards to stop smoking.

These data also provide a best estimate for the increase in birth weight associated with smoking cessation during pregnancy because of financial rewards of 206.00 g (95% CI, −69.12 to 481.14 g), or 6.1% of average birth weight (3.4 kg), which is less than the estimate of 387 g reduction for smokers from a recent cohort study.^1^ One explanation for this difference is that our estimate is from randomized trials where unrecognized unmeasured confounding is likely to be equally distributed between intervention and control groups, whereas unmeasured confounding will still be present using a cohort design. Furthermore, our estimate relates only to those women who are able to stop smoking as a result of the offer of financial rewards. There is a subset of women who are able to quit without this intervention. It is not known (and cannot be known) what their children’s birth weights would have been had they continued to smoke. These women are the most motivated (dubbed *independent quitters*),^12^ and their neonates have greater birth weight than those of women who only quit due to the financial rewards. They likely adopted other lifestyle changes during pregnancy, such as dietary changes, which may confer additional benefits above those from stopping smoking.

Even though this result is statistically significant, is it important?^26^ The most valuable consequence of this result is confirmation that increased smoking cessation, when women are offered financial rewards to stop smoking during pregnancy, is real. The findings are not due to gaming of the outcome measure, such as not smoking for 24 hours before outcome measurement using a carbon monoxide breath test.^9,27^ The improvement in birth weight strongly suggests that prolonged smoking cessation during pregnancy has taken place. This birth weight improvement is also manifest as a reduction in SGA births by 17.70%, suggesting that there will be 1 fewer neonate born SGA for every 6 women who quit because of rewards. Confirmation of smoking cessation also validates the high cost-effectiveness of financial rewards in this health care setting, providing £2 (US $2.60) in health care savings for every £1 (US $1.30) extra spent on cessation support.^28^ The largest trial in the current meta-analysis was the most diverse as financial rewards were added to 7 very varied SSSs supporting pregnant women across 3 of the 4 UK countries.^29^ However, even the most pragmatic of trials may not reflect the real-world situation when financial rewards are added to current smoking cessation support.

A phase 4 before-and-after study has been undertaken in Glasgow, UK,^30^ where financial rewards £160 (inflation-adjusted value, US $235) were at the lower end of the levels used in the trials within this meta-analysis (eTable 2 in Supplement 1). Even with this lower reward, pregnant smokers accepting cessation support increased from 41% to 51% (*P* < .001), and carbon monoxide–verified cessation in late pregnancy increased from 8% to 11% (*P* = .03).^30^ The intervention was successfully integrated into current services and was cost-effective. Although workload increased with more pregnant smokers accepting support, this was likely offset by less effort required to engage with women to offer support.

Further phase 4 research including analysis of birth weight, for example embedded within the funded roll-out of financial rewards for smoking cessation in pregnancy in England,^31^ is needed to clarify the most efficient frequency and level of financial rewards to use. For research purposes, an additional reliable measure of combustible tobacco use to verify cessation, with carbon monoxide–negative breath tests, is needed as widespread use of nicotine dispensing e-cigarettes over the last 10 years has undermined verification of smoking cessation by salivary cotinine.

### Strengths and Limitations

The strength of this study is use of an important health outcome—birth weight—that has not been biased by the rewards process, as it is a routine measurement in all jurisdictions. The main limitation of this study is the total sample size. In a randomized trial of the offer of financial rewards to stop smoking during pregnancy, only a minority of women will alter their behavior as a result of the intervention. The majority will either continue to smoke, regardless of the rewards on offer, or would have stopped anyway, so the association with birth weight at a population level will be heavily diluted. As an illustration, for a single trial to have 80% power at 5% significance to detect a mean 46-g difference in birth weight between groups, assuming an SD of 0.5 kg, a total sample size of 3712 participants would be required. Despite combining data from 8 trials, our maximum combined sample size was 2351.

## Conclusions

Financial rewards are being rolled out across England to help pregnant smokers to quit during pregnancy and to stay quit once their child is born after a published recommendation.^31^ Policymakers can be reassured that adding financial rewards to pregnancy smoking cessation support will result in a biochemically measured increase in smoking cessation associated with increased birth weight and an overall reduction in health care costs.

## Notes

Supplement 1. **eMethods**. Search strategy and selection criteria **eResults.** PRISMA diagram with additional 10 studies from extending the search by Kock^8^ from 17th November 2022 to 5th December 2023
eReferences
**eAppendix 1.** Table of excluded studies from Kock systematic review update searching from 17th November 2022 to 5th December 2023 **eAppendix 2.** GRADE certainty of evidence table **eTable 1.** Pooled estimates of the intention-to-treat (ITT) effects of the offer of financial rewards, and complier average causal effect (CACE) effects of smoking cessation, on study outcomes **eTable 2.** Maximum level of financial incentive and effectiveness of intervention **eFigure 1.** CACE estimates of smoking cessation during pregnancy on birth weight – sensitivity analysis **eFigure 2.** ITT estimates of the effect of financial rewards for smoking cessation on low birth weight **eFigure 3.** CACE estimates of smoking cessation during pregnancy on low birth weight **eFigure 4.** ITT estimates of the effect of financial rewards for smoking cessation on birth weight z-score **eFigure 5.** CACE estimates of smoking cessation during pregnancy on birth weight z-score **eFigure 6.** Sensitivity analyses of birth weight and low birth weight **eFigure 7.** Sensitivity analyses of birth weight z-score and SGA **eFigure 8.** Sensitivity analyses of birth weight and low birth weight **eFigure 9.** Sensitivity analyses of birth weight z-score and SGA

Supplement 2.
Data Sharing Statement
