# Supplementary material for: Financial Rewards for Smoking Cessation During Pregnancy and Birth Weight: A Meta-Analysis
Source: JAMA Netw Open. 2025 Mar 6;8(3):e250214. doi: 10.1001/jamanetworkopen.2025.0214 (PMC11886724; doi:10.1001/jamanetworkopen.2025.0214)
Supplement: Supplement 1. — eMethods. Search strategy and selection criteria eResults. PRISMA diagram with additional 10 studies from extending the search by Kock8 from 17th November 2022 to 5th December 2023 eReferences eAppendix 1. Table of excluded studies from Kock systematic review update searching from 17th November 2022 to 5th December 2023 eAppendix 2. GRADE certainty of evidence table eTable 1. Pooled estimates of the intention-to-treat (ITT) effects of the offer of financial rewards, and complier average causal effect (CACE) effects of smoking cessation, on study outcomes eTable 2. Maximum level of financial incentive and effectiveness of intervention eFigure 1. CACE estimates of smoking cessation during pregnancy on birth weight – sensitivity analysis eFigure 2. ITT estimates of the effect of financial rewards for smoking cessation on low birth weight eFigure 3. CACE estimates of smoking cessation during pregnancy on low birth weight eFigure 4. ITT estimates of the effect of financial rewards for smoking cessation on birth weight z-score eFigure 5. CACE estimates of smoking cessation during pregnancy on birth weight z-score eFigure 6. Sensitivity analyses of birth weight and low birth weight eFigure 7. Sensitivity analyses of birth weight z-score and SGA eFigure 8. Sensitivity analyses of birth weight and low birth weight eFigure 9. Sensitivity analyses of birth weight z-score and SGA [file jamanetwopen-e250214-s001.pdf]

# Supplemental Online Content

Tappin D, Lee J, McConnachie A, et al. Financial rewards for smoking cessation during pregnancy and birth weight: a meta-analysis. *JAMA Netw Open*. 2025;8(3):e250214. doi:10.1001/jamanetworkopen.2025.0214

**eMethods.** Search Strategy and selection criteria
**eResults.** PRISMA diagram
**eReferences**
**eAppendix 1.** Table of excluded studies from Kock systematic review update searching from 17th November 2022 to 5th December 2023
**eAppendix 2.** GRADE certainty of evidence table
**eTable 1.** Pooled estimates of the Intention-To-Treat (ITT) effects of the offer of financial rewards, and Complier Average Causal Effect (CACE) effects of smoking cessation, on study outcomes
**eTable 2.** Maximum level of financial incentive and effectiveness of intervention
**eFigure 1.** CACE Estimates of Smoking Cessation During Pregnancy on Birth Weight – Sensitivity Analysis
**eFigure 2.** ITT Estimates of the Effect of Financial Rewards for Smoking Cessation on Low Birth Weight
**eFigure 3.** CACE Estimates of Smoking Cessation During Pregnancy on Low Birth Weight
**eFigure 4.** ITT Estimates of the Effect of Financial Rewards for Smoking Cessation on Birth Weight Z-Score
**eFigure 5.** CACE Estimates of Smoking Cessation During Pregnancy on Birth Weight Z-Score
**eFigure 6.** Sensitivity Analyses of Birth Weight and Low Birth Weight
**eFigure 7.** Sensitivity Analyses of Birth Weight Z-Score and SGA
**eFigure 8.** Sensitivity Analyses of Birth Weight and Low Birth Weight
**eFigure 9.** Sensitivity Analyses of Birth Weight Z-Score and SGA

This supplemental material has been provided by the authors to give readers additional information about their work.

© 2025 Tappin D et al. *JAMA Network Open*.

**eMethods. Search Strategy and selection criteria**
As outlined by Kock et al[8], 'Medline, American Psychological Association PsycInfo, Embase, Cochrane (the Cochrane Central Register of Controlled Trials, the Cochrane Tobacco Addiction Group Specialized Register and the Cochrane Database of Systematic Reviews), and PubMed were searched from their inception until 5th December 2023 for published reports of RCTs or quasi-experimental or pragmatic trials (Cochrane, 2017) of incentives for abstinence from substance use among pregnant women. Only trials using an experimental design that will allow treatment effects to be attributed to the reward intervention were included.' As described, Kock et al[8] originally planned to include RCTs of incentives for abstinence from substances other than tobacco, but due to a small number of studies (only two returned after screening), these were excluded and the review focused solely on interventions for tobacco smoking cessation.

The following search terms for published literature on contingency management interventions for abstinence from substance use during pregnancy were used.

Study design: RCT or randomi?ed controlled trial or trial or randomi?ed or controlled clinical trial or pragmatic clinical trial (title or abstract or keyword)
Contingency management interventions: contingency management or incentive or financial incentive or voucher (title or abstract or keyword)
Pregnant women: Pregnan* (title or abstract or keyword)
Substance use: cigarette or smoking or smok* or nicotine or opioid or opiate or drug or stimulant or cocaine or meth* or amphetamine or alcohol or dependence or substance or substance use*'

**Types of study to be included**
Randomized controlled trials (RCTs) allocating individuals to intervention or to control conditions.

**Types of interventions**
Incentive schemes to reward participants for validated cessation and abstinence in smoking cessation programmes. Control groups could be usual care or a smoking cessation intervention similar to that provided in the experimental group, but without incentives. Studies comparing two interventions providing incentives, but which varied by the amount or type of incentive, or where intervention incentives were contingent on smoking cessation and control incentives were not contingent on smoking cessation, were also eligible.

**Condition or domain being studied**
Pregnancy and childbirth: Smoking cessation intervention during pregnancy

**Participants/population**
Pregnant women who are current smokers

**Intervention(s), exposure(s)**
The offer of financial incentives for smoking cessation during pregnancy in addition to routine stop smoking support

**Comparator(s)/control**
Routine stop smoking support

**Context**
Pregnant women who smoke tobacco usually in the context of maternity antenatal clinics but not excluding other contexts for the care of pregnant smokers

**Main outcome(s)**
Infant birth weight (in grams), gestation at birth (usually calculated from the date of the start of the Last Menstrual Period) and smoking cessation (biochemically verified) towards the end of pregnancy.

**Measures of effect**
Mean birth weight increase with the offer of financial incentives for smoking cessation in 4 groups for each included trial: 1. randomized to incentives for those who quit towards the end of pregnancy and 2. randomized to incentives who did not quit; 3. randomized to no incentives who quit towards the end of pregnancy and 4. randomized to no incentives who did not quit. Numbers in each group. Standard Deviation of birth weight for each group. Number of babies born in each group weighing less than 2500g.

© 2025 Tappin D et al. *JAMA Network Open*.

**Additional outcome(s)**
We will request data from each trial on infants born small for gestational age (SGA) in the 4 groups above by asking each trial to provide data for mean and standard deviation Weight for Gestational Age z-score using the app: http://intergrowth21.ndog.ox.ac.uk/ and numbers in each group below the 10th percentile for gestational age [z-score < (-1.2816)].

**Measures of effect**
Effect measure will be Birth Weight for Gestational Age z score (using mean and Standard deviation z-score) and number below 10th percentile birth weight for gestational age [Small for Gestational Age: z-score < (-1.2816)] in each of the 4 groups.

## eResults
PRISMA diagram with additional 10 studies from extending the search by Kock[8] from 17th November 2022 to 5th December 2023.

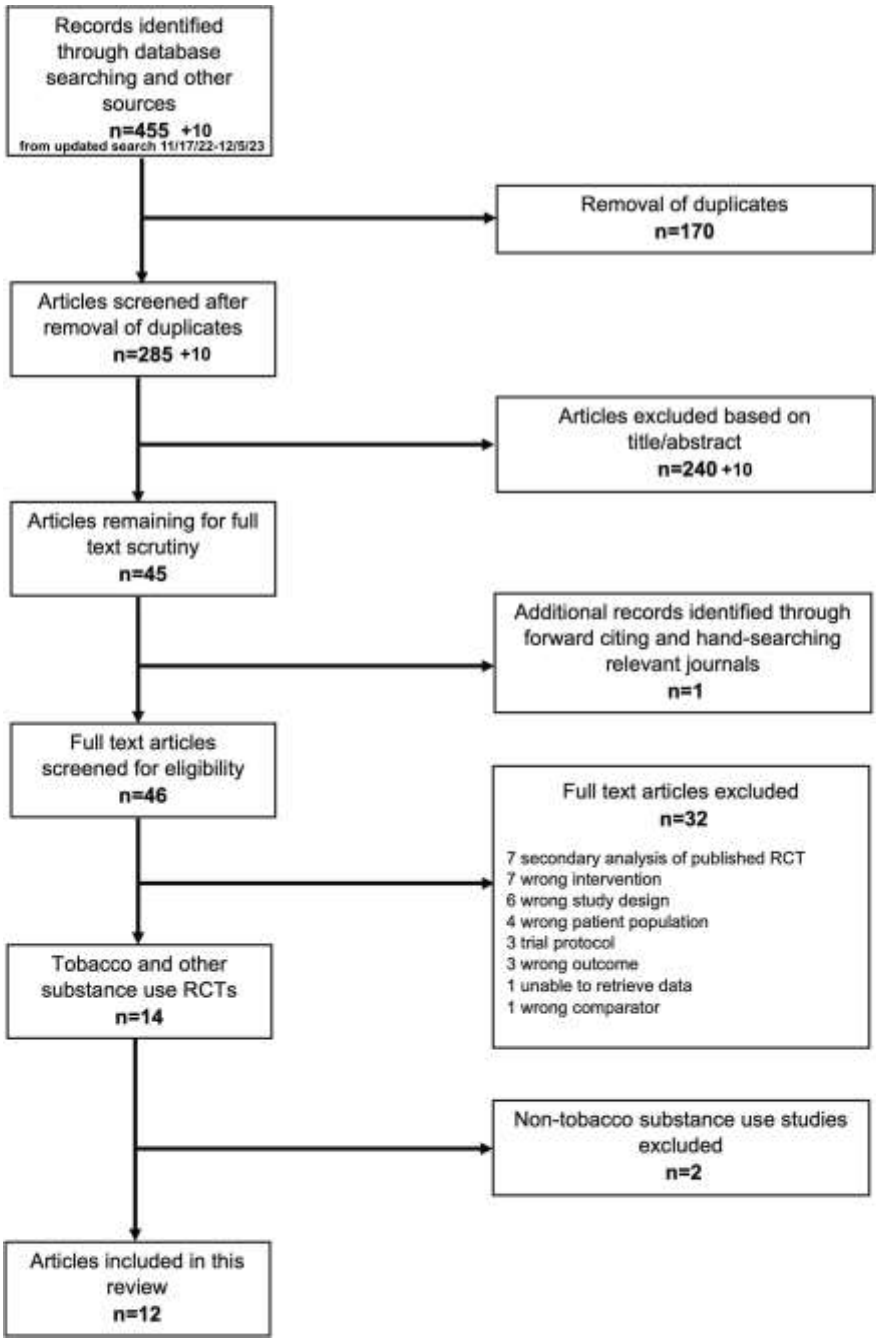


© 2025 Tappin D et al. *JAMA Network Open*.

## eReferences


8. Kock LS, Erath TG, Coleman SRM, Higgins ST, Heil SH. Contingency management interventions for abstinence from cigarette smoking in pregnancy and postpartum: A systematic review and meta-analysis. Prev Med 2023;176:107654. doi: 10.1016/j.ypmed.2023.107654.
9. Tappin D, Bauld L, Purves D, et al. Financial rewards for smoking cessation in pregnancy: randomised controlled trial. BMJ. 2015;350:h134. doi:10.1136/bmj.h134
18. Tappin D, Kock L, McConnachie A, Bauld L, Lee J. Financial incentives for smoking cessation during pregnancy: updating PROSPERO 2022 CRD42022372291 and re-focusing the outcome to birth weight. PROSPERO 2024 CRD42024494262. https://www.crd.york.ac.uk/prospero/display_record.php?ID=CRD42024494262. Accessed December 8, 2024.
19. Tappin D, Sinclair L, Kee F, et al. Effect of financial voucher rewards provided with UK stop smoking services on the cessation of smoking in pregnant women (CPIT III): pragmatic, multicentre, single blinded, phase 3, randomised controlled trial. BMJ. 2022;379:e071522. doi: 10.1136/bmj-2022-071522.
20. Berlin I, Berlin N, Malecot M, Breton M, Jusot F, Goldzahl L. Financial rewards for smoking cessation in pregnancy: multicentre randomised controlled trial. BMJ. 2021;375:e065217. doi: 10.1136/bmj-2021-065217.
21. Heil SH, Higgins ST, Bernstein IM, et al. Effects of voucher-based rewards on abstinence from cigarette smoking and fetal growth among pregnant women. Addiction. 2008;103(6):1009–1018. doi: 10.1111/j.1360-0443.2008.02237.x.
22. Higgins ST, Washio Y, Lopez AA, et al. Examining two different schedules of financial rewards for smoking cessation among pregnant women. Prev Med. 2014;68:51–57. doi: 10.1016/j.ypmed.2014.03.024.
23. Higgins ST, Nighbor TD, Kurti AN, et al. Randomized controlled trial examining the efficacy of adding financial rewards to best practices for smoking cessation among pregnant and newly postpartum women. Prev Med. 2022;165:107012. doi: 10.1016/j.ypmed.2022.107012.
24. Tuten M, Fitzsimons H, Chisolm MS, Nuzzo PA, Jones HE. Contingent rewards reduce cigarette smoking among pregnant, methadone-maintained women: results of an initial feasibility and efficacy randomized clinical trial. Addiction. 2012;107(10): 1868–1877. doi: 10.1111/j.1360-0443.2012.03923.x.
25. Ondersma SJ, Svikis DS, Lam PK, Connors-Burge VS, Ledgerwood DM, Hopper JA. A randomized trial of computer-delivered brief intervention and low-intensity contingency management for smoking during pregnancy. Nicotine Tob Res. 2012;14(3):351-360. doi: 10.1093/ntr/ntr221.


© 2025 Tappin D et al. *JAMA Network Open*.

**eAppendix 1: Table of excluded studies from Kock systematic review update searching from 17th November 2022 to 5th December 2023**

| No. | Reference | Summary comment for exclusion |
|---|---|---|
| **1** | Martin K, Dziva Chikwari C, Dauya E et al. Financial rewards to improve uptake of partner services for sexually transmitted infections in Zimbabwe antenatal care: protocol for a cluster randomised trial [version 2; peer review: 3 approved, 1 approved with reservations]. Wellcome Open Res 2023, 8:263 https://doi.org/10.12688/wellcomeopenres.19199.2 | Wrong outcome and intervention target |
| **2** | Chamie G, Hahn JA, Kekibiina A, Emenyonu NI, Beesiga B, Marson K, Fatch R, Lodi S, Adong J, Thirumurthy H, McDonell M, Gandhi M, Bryant K, Havlir DV, Kamya MR, Muyindike WR Financial rewards for reduced alcohol use and increased isoniazid adherence during tuberculosis preventive therapy among people with HIV in Uganda: an open-label, factorial randomised controlled trial. Lancet Glob Health 2023;11(12):e1899-910. https://doi.org/10.1016/S2214-109X(23)00430-8 | Wrong outcome, population and intervention target |
| **3** | Jackson MA, Brown AL, Baker AL, Bonevski B, Haber P, Bonomo Y, Blandthorn J, Attia J, Perry N, Barker D, Gould GS and Dunlop AJ (2023) Tobacco treatment incorporating contingency management, nicotine replacement therapy, and behavioral counseling for pregnant women who use substances: a feasibility trial. Front. Psychiatry 14:1207955. doi: 10.3389/fpsyt.2023.1207955 | Wrong study design: fnon-randomized feasibility trial |
| **4** | Kock LS, Erath TG, Coleman SRM, Higgins ST, Heil SH. Contingency management interventions for abstinence from cigarette smoking in pregnancy and postpartum: A systematic review and meta-analysis. Prev Med 2023;176:107654. doi: 10.1016/j.ypmed.2023.107654. | Wrong Design: Systematic Review |
| **5** | (2023), Poster Abstract. Alcohol Clin Exp Res, 47: 148-573. https://doi.org/10.1111/acer.15071 | Wrong outcome, population and study design |
| **6** | McMeekin N, Sinclair L, Robinson-Smith L, Mitchell A, Bauld L, Tappin DM, et al. Financial rewards for quitting smoking in pregnancy: Are they cost-effective? Addiction 2023;118(8):1445–56. https://doi.org/10.1111/add.16176 | Wrong design: Cost-effectiveness analysis of previously published RCT |

© 2025 Tappin D et al. *JAMA Network Open*.

| | | |
|---|---|---|
| **7** | Evemy, C. G., Kurti, A. N., Skelly, J. M., Medina, N. A., & Higgins, S. T. (2023). Examining the latent factor structure of a hypothetical cigarette purchase task among pregnant women. Experimental and Clinical Psychopharmacology. 2023;31(1):23–8. https://doi.org/10.1037/pha0000571 | Wrong study design, intervention and outcome. |
| **8** | Abstracts from the 7th UK Congress on Obesity 2022. Int J Obes 2023;47 (Suppl 1), 1–33. https://doi.org/10.1038/s41366-023-01306-4 | Wrong outcome and intervention target |
| **9** | Hensen B, Floyd S, Phiri MM, Schaap A, Sigande L, Simuyaba M, et al. The impact of community-based, peer-led sexual and reproductive health services on knowledge of HIV status among adolescents and young people aged 15 to 24 in Lusaka, Zambia: The Yathu Yathu cluster-randomised trial. PLoS Med 2023;20(4): e1004203. https://doi.org/10.1371/journal.pmed.1004203 | Wrong outcome, population and intervention target |
| **10** | Bardou M, Meunier-Beillard N, Godard-Marceau A on behalf of the NAITRE Study group, et al. Women and health professionals' perspectives on a conditional cash transfer programme to improve pregnancy follow-up: a qualitative analysis of the NAITRE randomised controlled study. BMJ Open 2023;13:e067066. doi: 10.1136/bmjopen-2022-067066 | Wrong study design and study population. |

© 2025 Tappin D et al. *JAMA Network Open*.

## eAppendix 2:GRADE certainty of evidence table

| Grade criteria | Rating | Comments (reason for downgrading/upgrading) | Certainty of evidence |
|---|---|---|---|
| **Outcome: Birth Weight** | | | |
| Study design | RCTs (starts as high) | RCTs | Moderate +++0 |
| Risk of bias | - | Birth weight was available in only 8 of 12 trials in the Kock review (8). For 7 of 8 smoking cessation increased and mean birth weight increased. For one of 8 (25) smoking cessation reduced and mean birth weight reduced. There is therefore a consistent association. This current review attempted to contact all 12 trials from Kock (8). For Donatelle (2 trials) (32) and Glover (33) contact proved impossible and Kurti (34) did not collect birth weight data. Donatelle and Kurti both showed an increase in smoking cessation with rewards likely leading to an increase in birth weight, Glover a small pilot trial showed no change in smoking cessation.<br>This makes bias from birth weight being unavailable in 4 of the 12 trials unlikely. | |
| Inconsistency | - | Low levels of heterogeneity ($I^2$ = 0%) in meta-analysis model. | |
| Indirectness | - | Trial populations are directly comparable (pregnant women who are currently smoking). Trials conducted in US, UK, and France. | |
| Imprecision | Serious (-1) | Mix of larger and smaller sample sizes. Not enough to calculate a precise effect estimate as the post-hoc calculated single trial sample size with 80% power and p<0.05 to show a 46g increase in birth weight is 3712. Our sample size is 2,351. However, upper and lower limits of effect estimate are consistent with a beneficial effect of reward interventions for infant birth weight. | |
| Publication bias | - | Exploration of publication bias through funnel plot was not possible as only 8 trials were included. The systematic review (8), on which this review is based, showed no evidence of publication bias using funnel plot and Egger's test (35). | |

© 2025 Tappin D et al. *JAMA Network Open*.

**eTable 1.** Pooled estimates of the Intention-To-Treat (ITT) effects of the offer of financial rewards, and Complier Average Causal Effect (CACE) effects of smoking cessation, on study outcomes. Pooled estimates derived from random effects models.

| **Outcome** | **Estimator** | **Analysis** | **Estimate (95% CI)** | ***P* Value** |
|---|---|---|---|---|
| Birth Weight (g) | Mean Difference | ITT | 49.7 (-12.49, 111.94) | .12 |
| | | CACE | 207.6 (-92.93, 508.09) | .18 |
| Birth Weight <2.5kg | Risk Difference | ITT | -0.70% (-4.71%, 3.35%) | .74 |
| | | CACE | -3.7% (-22.46%, 14.97%) | .69 |
| Birth Weight z-score | Mean Difference | ITT | 0.0 (-0.16, 0.20) | .84 |
| | | CACE | 0.3 (-0.39, 0.95) | .42 |
| Small for Gestational Age | Risk Difference | ITT | -2.6% (-7.79%, 2.52%) | .32 |
| | | CACE | -17.7% (-34.93%, -0.39%) | .05 |

**eTable 2**. Maximum level of financial incentive and effectiveness of intervention

| Trial | Maximum level of financial incentive payable antepartum ($US corrected for inflation from publication year to today) | Risk ratio of cessation towards end of pregnancy (95%CI) | Mean birth weight change (kg) (95%CI) |
|---|---|---|---|
| Berlin et al,[20] 2021 | 520 euros ($659 US) | 2.22 (1.29,3.81) | 0.05 (-0.06,0.15) |
| Heil et al,[21] 2008 | $1180 US dollars ($1722 US) | 4.05 (1.48,11.11) | 0.20 (-0.07,0.48) |
| Higgins et al,[22] 2014 | $1180 US ($1569 US) | 2.26 (1.10,4.65) | 0.13 (-0.13,0.38) |
| Higgins et al,[23] 2022 | $1180 US ($1263 US) | 4.21 (2.06,8.62) | 0.04 (-0.10,0.17) |
| Ondersma et al,[25] 2012 | $250 US ($343 US) | 0.78 (0.20,3.11) | -0.08 (-0.36,0.19) |
| Tappin et al,[9] 2015 | £400 ($690 US) | 2.63 (1.72,4.01) | 0.02 (-0.07,0.12) |
| Tappin et al,[19] 2022 | £400 ($555 US) | 2.17 (1.63,2.88) | 0.05 (-0.03,0.13) |
| Tuten et al,[24] 2012 | $736 US ($1008 US) | 20.72 (1.28,336.01) | 0.16 (-0.19,0.52) |

© 2025 Tappin D et al. *JAMA Network Open*.

| Source | Control Smoker | Control Quitter | Incentives Smoker | Incentives Quitter | CACE [95% CI] | Weight |
|---|---|---|---|---|---|---|
| Berlin et al,[20] 2021 | 3.04 | 3.40 | 3.02 | 3.34 | 0.11 [-0.58; 0.80] | 15.9% |
| Heil et al,[21] 2008 | 3.12 | 3.15 | 3.33 | 3.32 | 0.60 [-0.24; 1.45] | 10.5% |
| Higgins et al,[22] 2014 | 3.05 | 3.85 | 3.17 | 3.51 | 0.47 [-0.66; 1.60] | 5.9% |
| Higgins et al,[23] 2022 | 3.16 | 3.46 | 3.08 | 3.25 | -0.13 [-0.76; 0.51] | 18.8% |
| Ondersma et al,[25] 2012 | 2.84 | 3.40 | 2.86 | 2.95 | 1.24 [-3.34; 5.81] | 0.4% |
| Tappin et al,[19] 2022 | 3.08 | 3.49 | 3.07 | 3.46 | 0.31 [-0.19; 0.80] | 31.0% |
| Tappin et al,[9] 2015 | 3.08 | 3.59 | 3.05 | 3.43 | 0.15 [-0.51; 0.80] | 17.5% |
| **Fixed effect model** | | | | | **0.21 [-0.06; 0.48]** | **100.0%** |

Heterogeneity: $I^2 = 0\%$, $\tau^2 = 0.0351$, $p = 0.86$

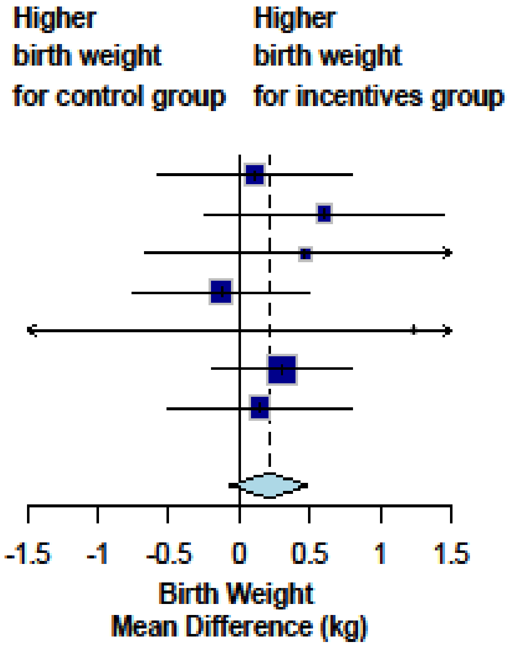


eFigure 1.  CACE Estimates of Smoking Cessation During Pregnancy on Birth Weight – Sensitivity Analysis

Forest plot showing the Complier Average Causal Effect (CACE) estimates of the effect of smoking cessation during pregnancy on birth weight (kg). The pooled effect was calculated using a fixed effect model. The size of data markers is proportional to the weight in the meta-analysis.

© 2025 Tappin D et al. *JAMA Network Open*.

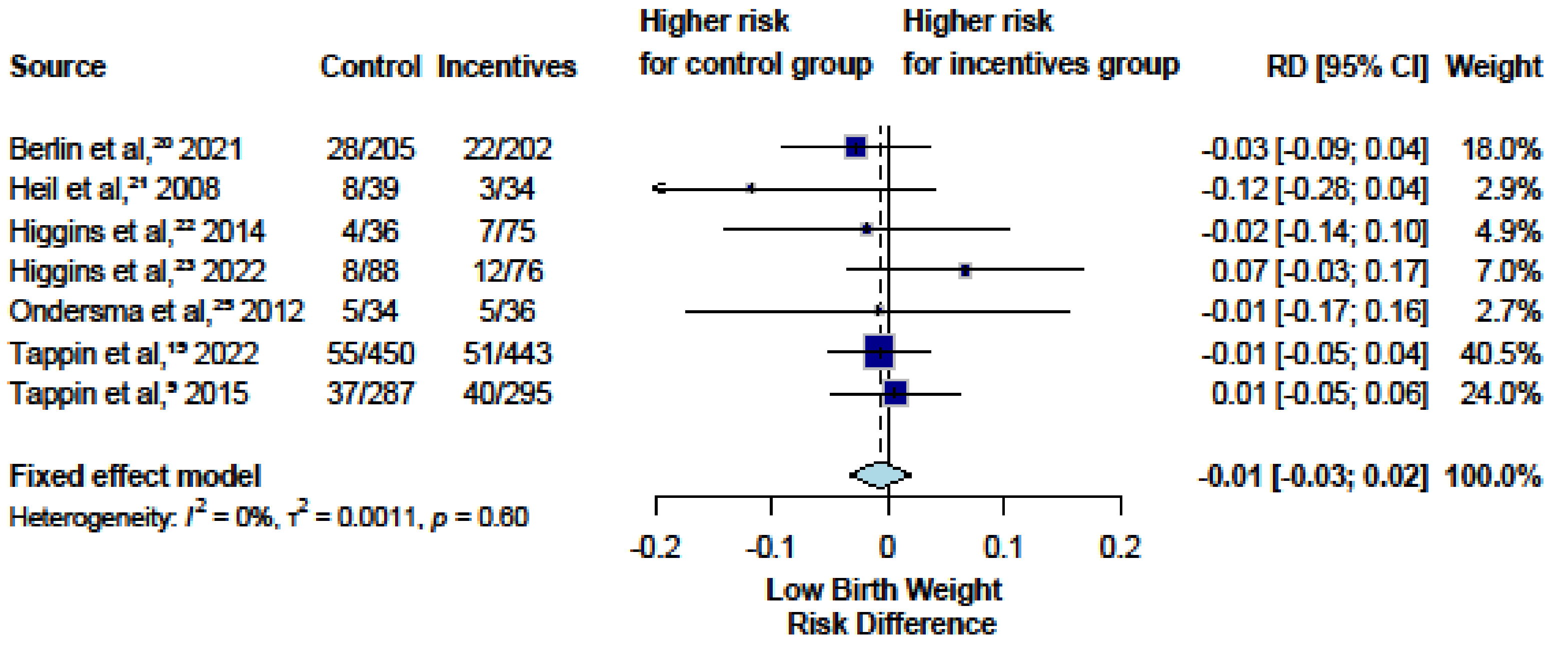


eFigure 2. ITT Estimates of the Effect of Financial Rewards for Smoking Cessation on Low Birth Weight

Forest plot showing the intention-to-treat (ITT) estimates of the effect of the offer of financial rewards for smoking cessation during pregnancy on low birth weight (<2.5kg), expressed as risk differences. The pooled effect was calculated using a fixed effect model. The size of data markers is proportional to the weight in the meta-analysis.

© 2025 Tappin D et al. *JAMA Network Open*.

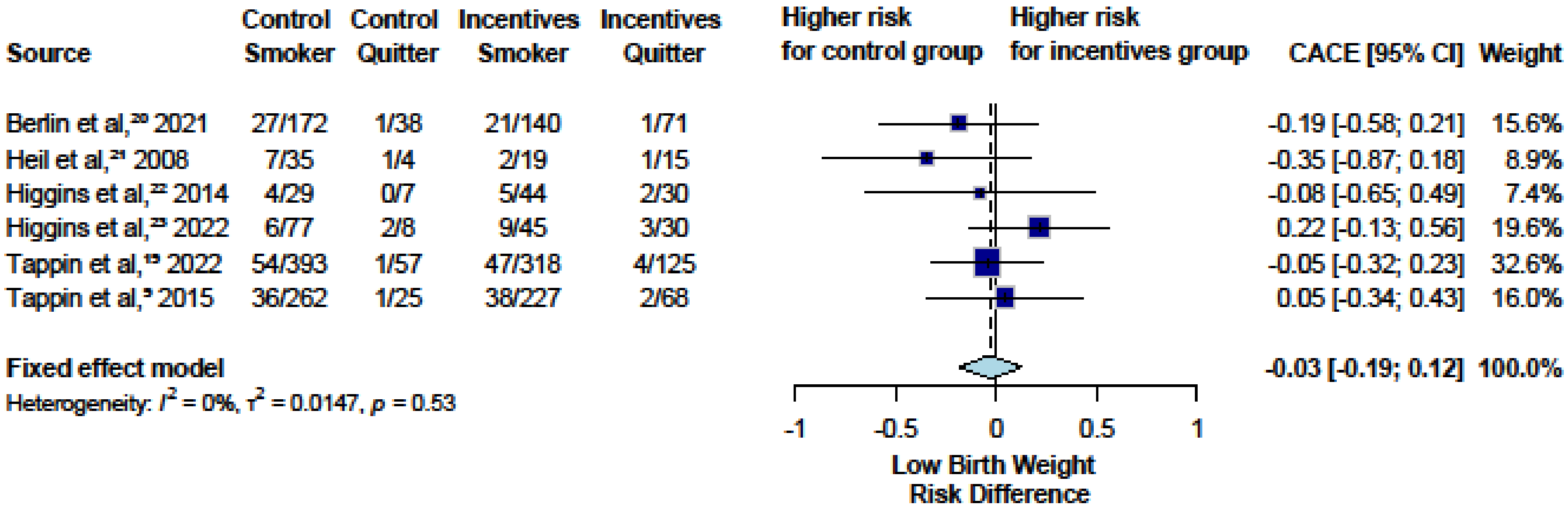


eFigure 3. CACE Estimates of Smoking Cessation During Pregnancy on Low Birth Weight

Forest plot showing the Complier Average Causal Effect (CACE) estimates of the effect of smoking cessation during pregnancy on low birth weight (<2.5kg), expressed as risk differences. The pooled effect was calculated using a fixed effect model. The size of data markers is proportional to the weight in the meta-analysis.

© 2025 Tappin D et al. *JAMA Network Open*.

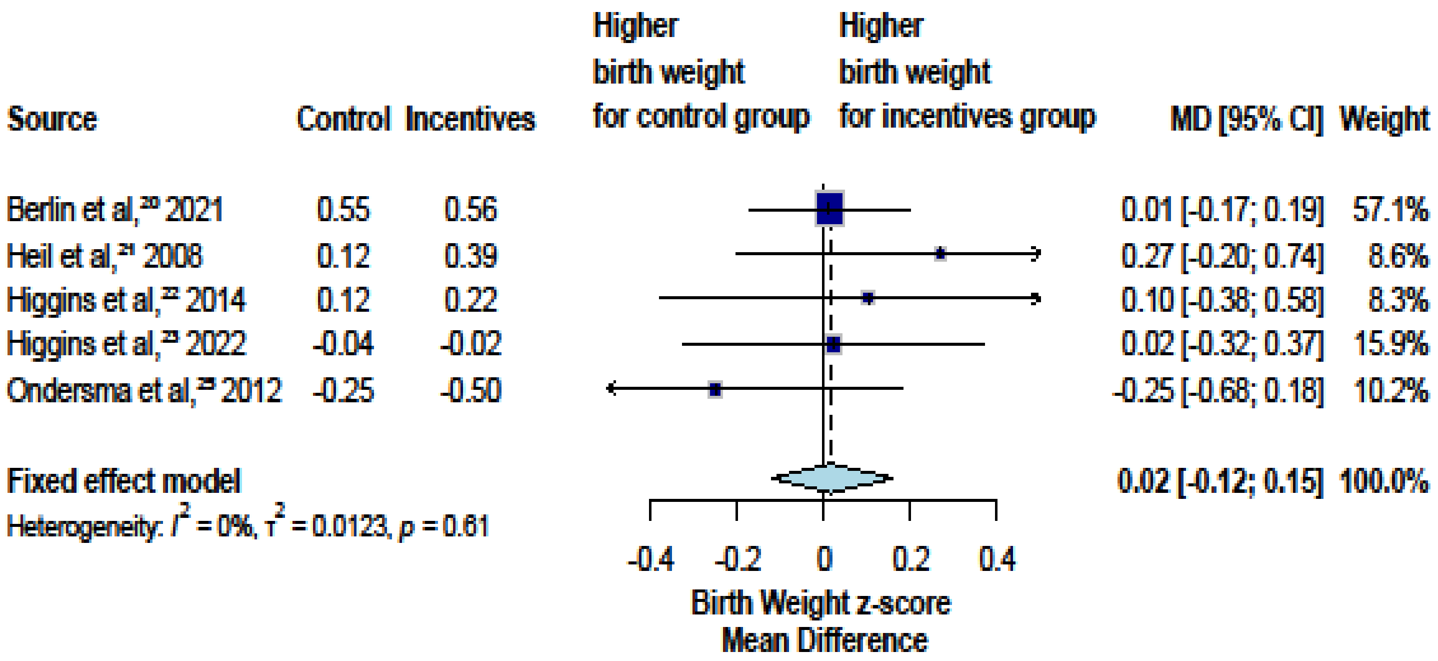


eFigure 4. ITT Estimates of the Effect of Financial Rewards for Smoking Cessation on Birth Weight Z-Score

Forest plot showing the intention-to-treat (ITT) estimates of the effect of the offer of financial rewards for smoking cessation during pregnancy on birth weight z-score (adjusted for sex and gestational age). The pooled effect was calculated using a fixed effect model. The size of data markers is proportional to the weight in the meta-analysis.

© 2025 Tappin D et al. *JAMA Network Open*.

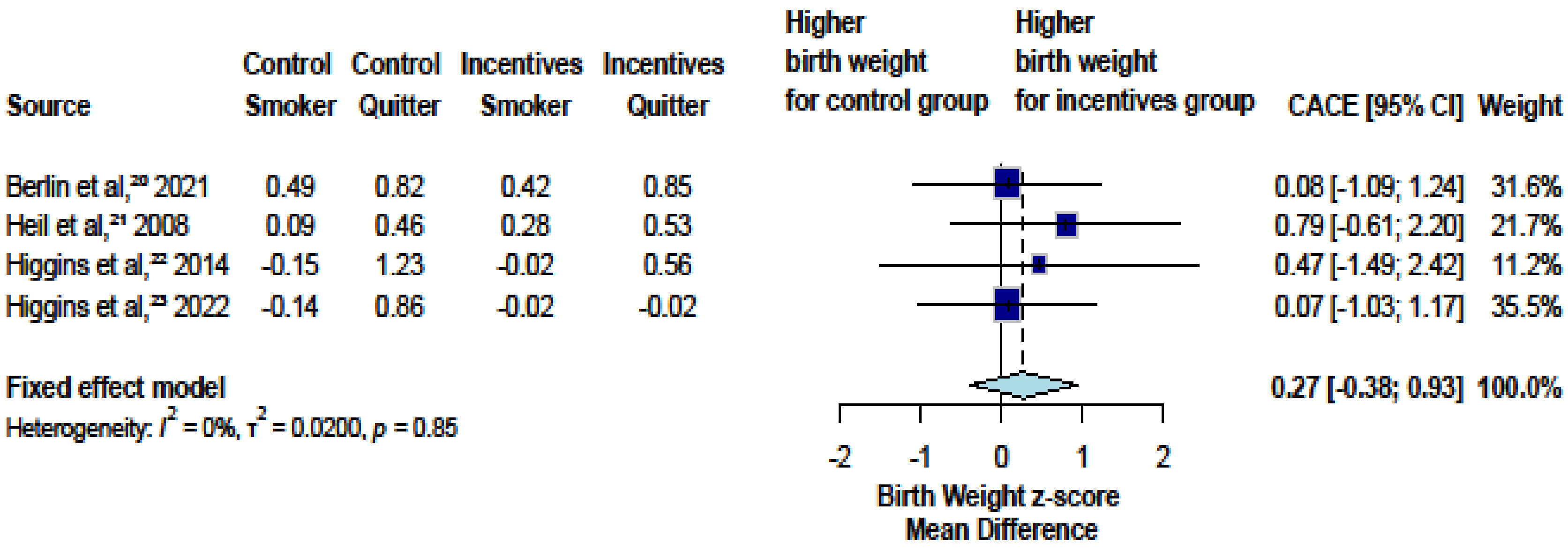


eFigure 5. CACE Estimates of Smoking Cessation During Pregnancy on Birth Weight Z-Score

Forest plot showing the Complier Average Causal Effect (CACE) estimates of the effect of smoking cessation during pregnancy on birth weight z-score (adjusted for sex and gestational age). The pooled effect was calculated using a fixed effect model. The size of data markers is proportional to the weight in the meta-analysis.

© 2025 Tappin D et al. *JAMA Network Open*.

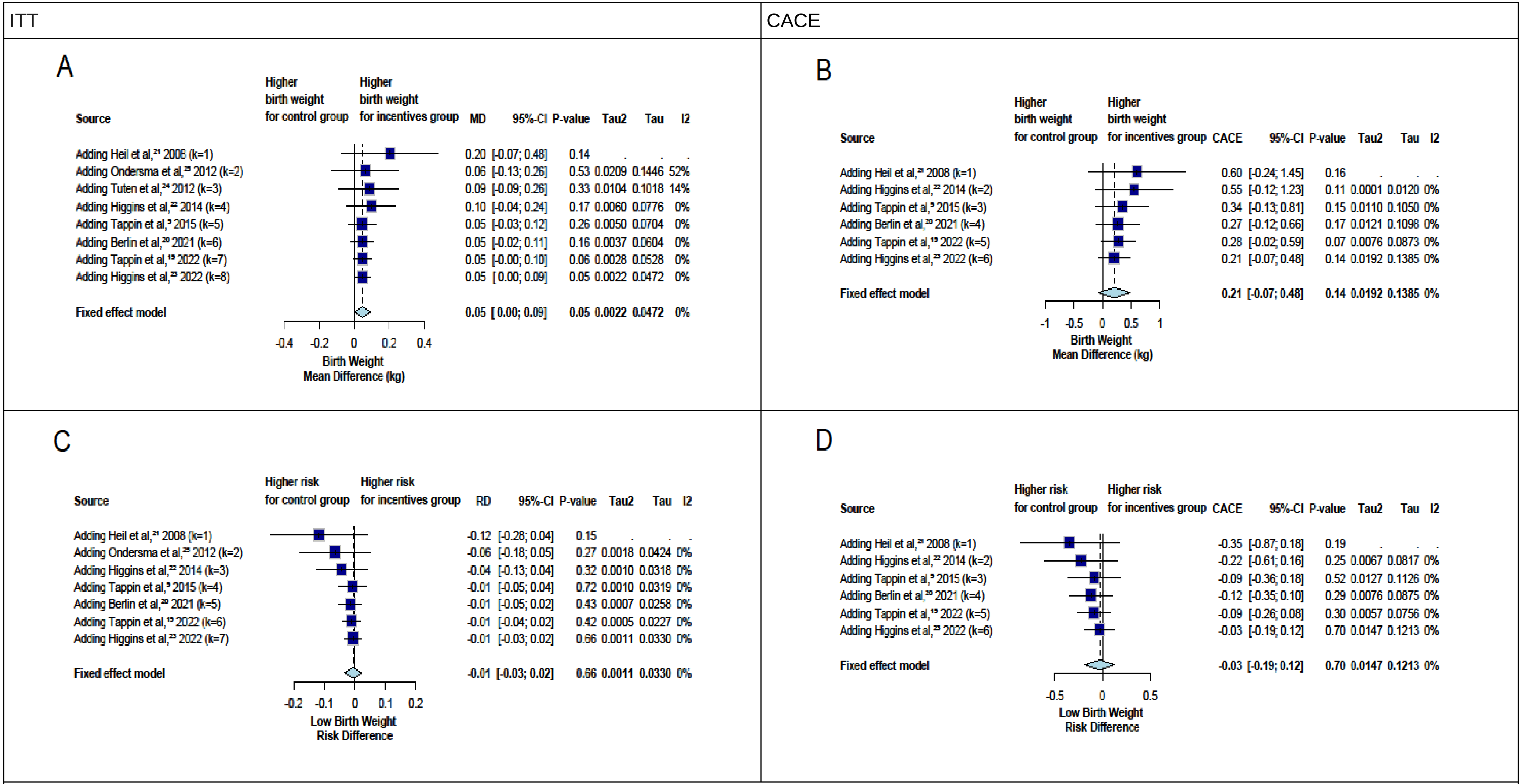


eFigure 6. Sensitivity Analyses of Birth Weight and Low Birth Weight

Forest plots showing cumulative meta-analyses of birth weight (top row) and low birth weight (<2500g, bottom row); Panels A and C show the intention-to-treat (ITT) analyses, representing the estimated difference between randomised groups. Panels B and D show the Complier Average Causal Effect (CACE), indicating the estimated causal effect of smoking cessation. Each plot shows the cumulative pooled estimate as results from each trial are added, in chronological order by publication date.

© 2025 Tappin D et al. *JAMA Network Open*.

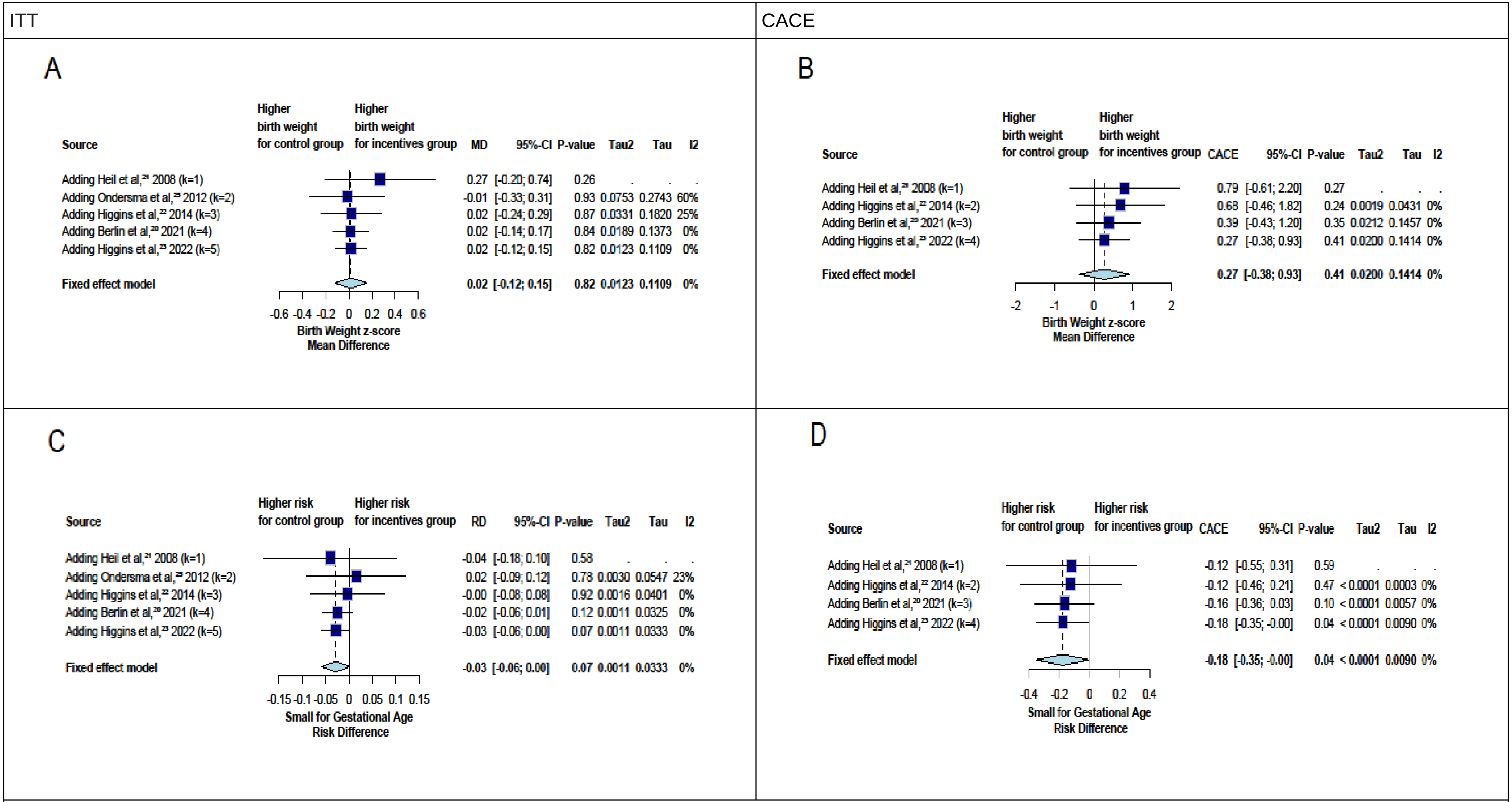


eFigure 7. Sensitivity Analyses of Birth Weight Z-Score and SGA

Forest plots showing cumulative meta-analyses of gestational age- and sex-adjusted birth weight z-score (top row), and small for gestational age, SGA (<10th percentile, bottom row); Panels A and C show the intention-to-treat (ITT) analyses, representing the estimated difference between randomised groups. Panels B and D show the Complier Average Causal Effect (CACE), indicating the estimated causal effect of smoking cessation. Each plot shows the cumulative pooled estimate as results from each trial are added, in chronological order by publication date.

© 2025 Tappin D et al. *JAMA Network Open*.

| ITT | CACE |
|---|---|

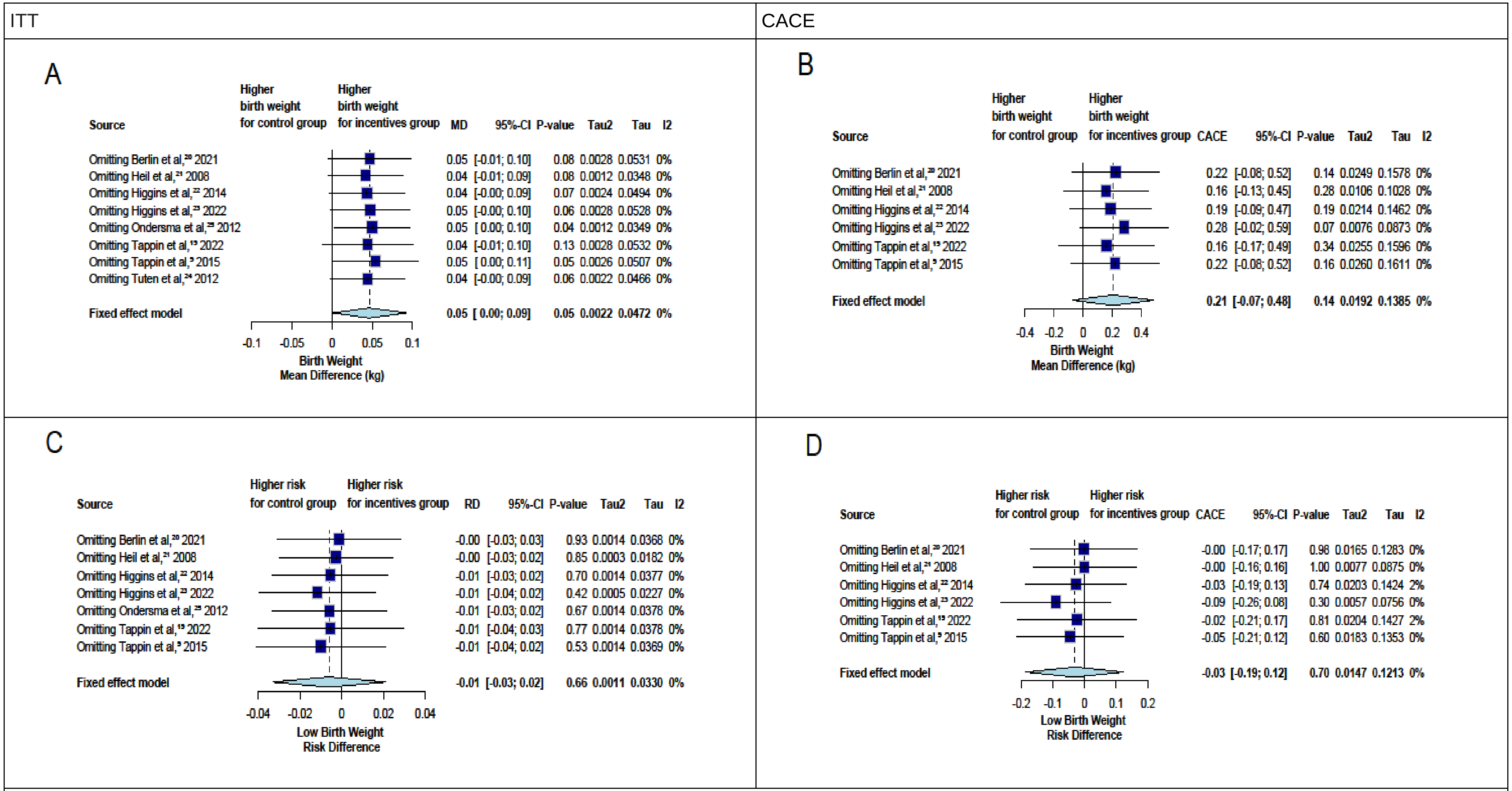


eFigure 8. Sensitivity Analyses of Birth Weight and Low Birth Weight

Forest plots showing leave-out-out meta-analyses of birth weight (top row) and low birth weight (<2500g, bottom row); Panels A and C show the intention-to-treat (ITT) analyses, representing the estimated difference between randomised groups. Panels B and D show the Complier Average Causal Effect (CACE), indicating the estimated causal effect of smoking cessation. Each plot shows the pooled estimate after the exclusion of each trial.

© 2025 Tappin D et al. *JAMA Network Open*.

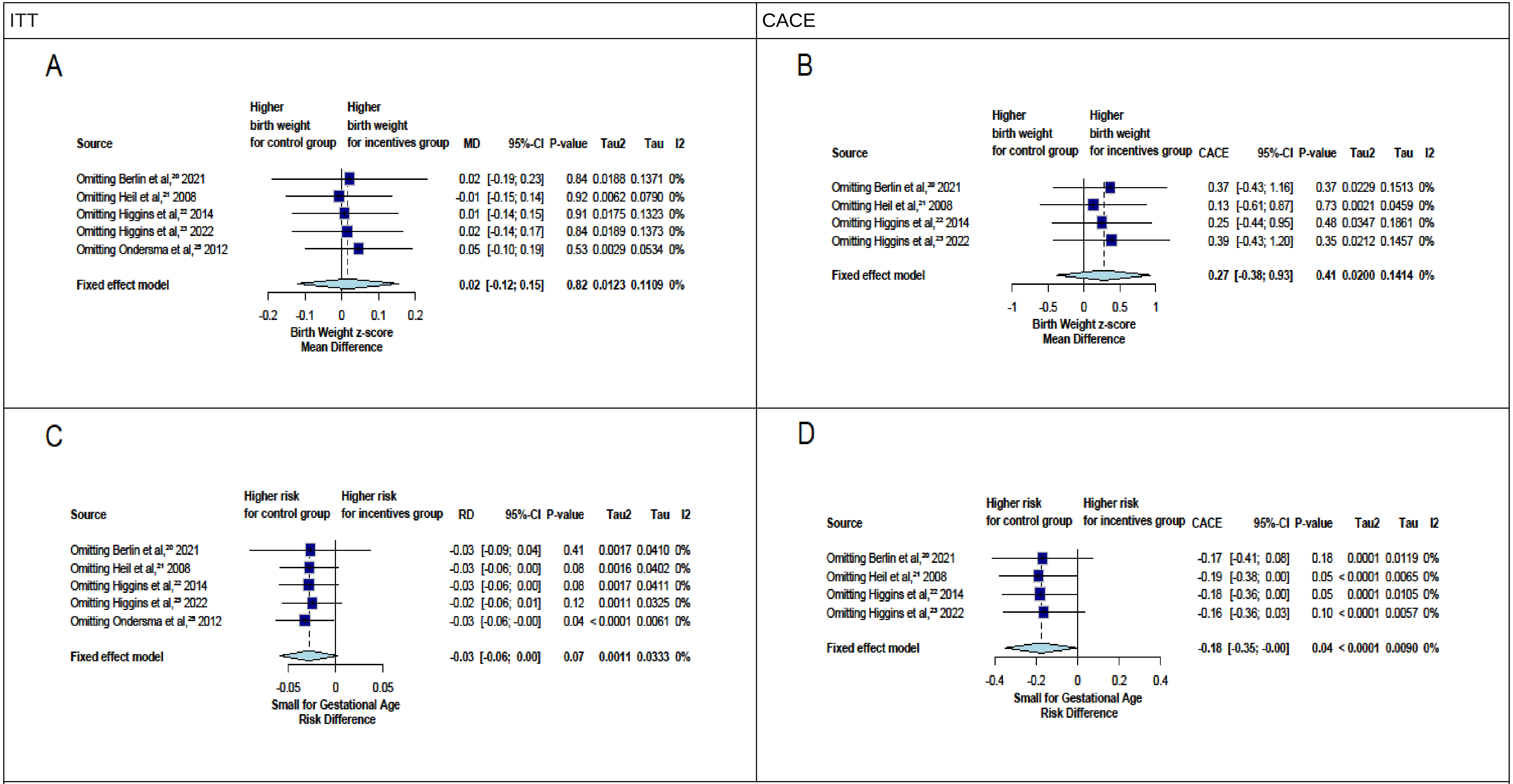


## eFigure 9. Sensitivity Analyses of Birth Weight Z-Score and SGA

Forest plots showing leave-one-out meta-analyses of gestational age- and sex-adjusted birth weight z-score (top row), and small for gestational age, SGA (<10th percentile, bottom row); Panels A and C show the intention-to-treat (ITT) analyses, representing the estimated difference between randomised groups. Panels B and D show the Complier Average Causal Effect (CACE), indicating the estimated causal effect of smoking cessation. Each plot shows the pooled estimate after the exclusion of each trial.

© 2025 Tappin D et al. *JAMA Network Open*.

© 2025 Tappin D et al. *JAMA Network Open*.
