# Supplementary material for: Financial Rewards for Smoking Cessation During Pregnancy and Birth Weight: A Meta-Analysis
Source: JAMA Netw Open. 2025 Mar 6;8(3):e250214. doi: 10.1001/jamanetworkopen.2025.0214 (PMC11886724; doi:10.1001/jamanetworkopen.2025.0214)
Supplement: Supplement 2. — Data Sharing Statement [file jamanetwopen-e250214-s002.pdf]

Data Sharing Statement

Tappin. Financial Rewards for Smoking Cessation During Pregnancy and Birth Weight. *JAMA Netw Open*. Published March 06, 2025. doi:10.1001/jamanetworkopen.2025.0214

Data
Data available: No

Additional Information
Explanation for why data not available: Individual patient data was not collected for this report
